# A Treatise on Human Physiology

**Published:** 1861-05

**Authors:** 


ART. VIII.—*A Treatise on Human Physiology; designed for the Use of Students and Practitioners of Medicine.* By JOHN C. DALTON, JR., M.D., Professor of Physiology and Microscopic Anatomy in the College of Physicians and Surgeons of New York, etc. 8vo., pp. 690. Blanchard & Lea: Philadelphia, 1861.

A SECOND edition of this deservedly popular work having been called for in the short space of two years, the author has supplied deficiencies, which existed in the former volume, and has thus more completely fulfilled his design of presenting to the profession a reliable and precise text-book, and one which we consider the best outline on the subject of which it treats in any language. Dealing more with the important facts and discoveries in physiological science, Dr. Dalton does not encumber his work with the discussion of unsettled questions or theoretical topics, but presents his readers with a clear and concise digest of all that is really worthy of being known. The additions in the present issue consist in the introduction of a chapter on the special senses; a chapter on imbibition, exhalation, and the functions of the lymphatic system; the rearrangement of the chapter on the cranial nerves; alterations in the sections on secretion, excretion, the circulation, and the functions of the digestive apparatus; considerations concerning the general properties of sensation and motion, as resident in the nervous system, and an account of some original experiments and conclusions relating to the function of the cerebellum.

The opinion, first advocated by Flourens, that the cerebellum was the centre of association or co-ordination of the different voluntary movements, has since been proved by different physiologists, by removing the whole or portions of that organ, when this power is lost. Dr. Dalton shows, however, that even when a large part of the cerebellum of a bird has been removed, the control over muscular contractions becomes re-established in the course of time; and that the organ remains in the same condition as immediately after its removal, no attempt having been made at its regeneration. This temporary disturbance of the co-ordinating power, the author regards as due to the sudden injury of the cerebellum as a whole, and not to the mere removal of a portion of its mass.

We have searched in vain in this volume for an account of the cerebral circulation and of the cerebro-spinal fluid, both of which play so important a part in the physiology of the nervous centres. Can it be that we have failed to find it, or that the distinguished author has not deemed these matters of sufficient consequence to bestow upon them even a passing notice in a purely elementary treatise?

